# Warfarin-Durian interaction: A case study bridging clinical outcomes and metabolic profiling through metabolomics

**DOI:** 10.1016/j.toxrep.2026.102232

**Published:** 2026-02-25

**Authors:** Natthapat Hiranchatchawal, Konwalin Wannaphueak, Piyapat Rattanasuwan, Prem Lertpongpipat, Giatgong Konguthaithip, Churdsak Jaikang, Preechaya Tajai

**Affiliations:** a Faculty of Medicine, Chiang Mai University, Chiang Mai 50200, Thailand; b Department of Forensic Medicine, Faculty of Medicine, Chiang Mai University, Chiang Mai 50200, Thailand; c Metabolomic Research Group for Forensic Medicine and Toxicology, Department of Forensic Medicine, Faculty of Medicine, Chiang Mai University, Chiang Mai 50200, Thailand

**Keywords:** ^1^H NMR metabolomics, Drug-food interaction, Metabolites, Purine metabolism, Tricarboxylic acid cycle

## Abstract

Warfarin-food interactions present significant challenges in achieving optimal anticoagulation; however, comprehensive research remains limited. This preliminary study explores possible mechanisms underlying the effects of durian consumption on warfarin efficacy, based on clinical observations of international normalized ratio (INR). A cross-sectional study was conducted in patients undergoing warfarin therapy who exhibited INR exceeding the therapeutic range after repeated durian consumption (one to three pieces per day for five days to one month). Plasma samples were analyzed using proton nuclear magnetic resonance (^1^H NMR)-based metabolomics to identify metabolic alterations. Patients who consumed durian exhibited a statistically significant increase in INR (3.93 ± 0.60) compared to the control group (2.48 ± 0.32, *p* = 0.0003). Sulfur-containing compounds were biomarkers of durian exposure, with hydrogen sulfide, trimethylsulfonium, and thiosulfate significantly increased in the warfarin-durian interaction group (*p* < 0.05). Metabolomic analysis revealed significant disruptions in purine metabolism and the tricarboxylic acid (TCA) cycle, with adenylosuccinic acid and fumaric acid identified as key biomarkers. The reduction in adenylosuccinic acid suggests impaired purine metabolism, while the increase in fumaric acid indicates TCA cycle dysregulation. This study is the first to utilize metabolomics to investigate warfarin-durian interactions, integrating clinical and metabolic insights, and highlights the potential of metabolomics in drug-food interaction research and patient safety.

## Highlights

• This is the first study to employ metabolomics in investigating the warfarin-durian interaction.
• Durian consumption may increase the risk of bleeding in patients taking warfarin.
• Sulfur-containing metabolites are potential exposure biomarkers of durian consumption.
• Metabolomics reveals disruptions in purine metabolism and the TCA cycle during the warfarin-durian interaction.
• ^1^H NMR metabolomics holds promise for providing a more comprehensive understanding of drug-food interactions.

## Graphical Abstract

**Figure:**
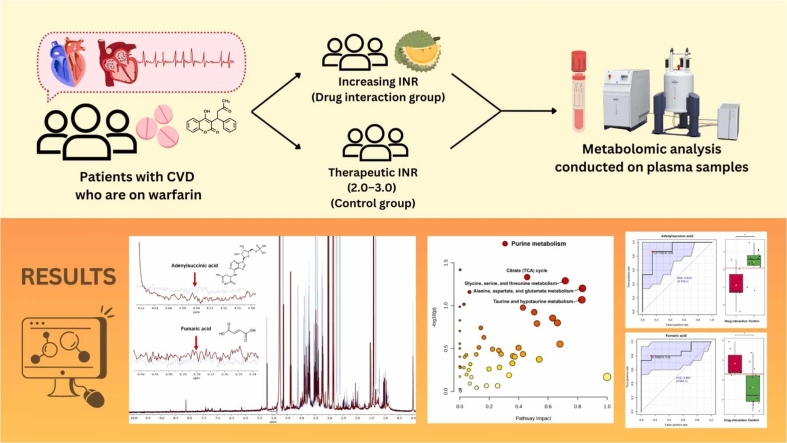

## 1 Introduction

Warfarin remains an important oral anticoagulant in contemporary clinical practice, particularly in specific patient populations and healthcare settings [1], [2], [3]. While direct oral anticoagulants (DOACs) are increasingly prescribed for the treatment of venous thromboembolism and for the prevention of embolic stroke in patients with atrial fibrillation, warfarin continues to play a key role in specific clinical settings where DOACs are not recommended, or their use is limited [4], [5], [6]. Additionally, warfarin remains the anticoagulant of choice for patients with mechanical prosthetic heart valves to reduce the risk of thromboembolic events [4], [5], [6]. Warfarin exhibits anticoagulant properties by inhibiting vitamin K-dependent clotting factors, thereby preventing clot formation [1], [2], [3]. While warfarin is highly effective, its use is challenged by its narrow therapeutic window, requiring careful dosing to maintain optimal anticoagulation while minimizing the risk of bleeding [7], [8]. The use of warfarin requires close monitoring of the International Normalized Ratio (INR), a standardized measure derived from prothrombin time (PT), to ensure it remains within the therapeutic range and to prevent complications [2]. Warfarin is a racemic mixture of R- and S-enantiomers, and its metabolism primarily relies on cytochrome P450 (CYP) enzymes, particularly CYP2C9, CYP2C19, CYP1A2, and CYP3A4 [2], [9], [10]. The primary complication of warfarin therapy is the risk of over-anticoagulation, which can result from various factors [2], [3]. Among these, genetic polymorphisms in CYP enzymes play a major role by reducing the metabolic clearance of warfarin, thereby increasing its plasma concentration [2], [11]. Additionally, warfarin dosing is influenced by various patient-specific factors, including diet, liver function, comorbidities, lifestyle, and concurrent medications [9], [12]. While the effects of warfarin interactions with other drugs are well-established, research on its interactions with fruits remains limited [10].

The durian (*Durio zibethinus* L.) is a tropical fruit native to Southeast Asia, including Thailand, and belongs to the Malvaceae family [13], [14]. Known as the king of fruits, it is prized for its rich, sweet, and creamy flavor, and its strong, distinctive aroma [15]. This leads many people, especially Thais, to consume durian, including patients who are currently on warfarin therapy [16]. However, limited clinical studies have suggested a potential association between durian consumption and INR out of the therapeutic range [10], [12]. Durian pulp is rich in valuable bioactive compounds, including polyphenols, such as flavonoids (flavanones, flavonols, flavones, flavanols, and anthocyanins), phenolic acids (cinnamic acid and hydroxybenzoic acid), tannins, and other bioactive components like carotenoids and ascorbic acid [13]. Polyphenols are known to inhibit CYP enzymes, particularly CYP2C9, a key enzyme involved in warfarin metabolism [17]. In addition, durian pulp contains particularly high levels of sulfur-based compounds, which are primarily responsible for the characteristic volatile aroma of the fruit [18], [19]. Major sulfur volatiles identified in durian include hydrogen sulfide, methanethiol, propane-1-thiol, diethyl sulfide, dimethyl thioester, ethane, ethyl hydro disulfide, diethyl disulfide, and diethyl trisulfide [18]. Several studies have demonstrated that sulfur-containing compounds and their sulfate metabolites can inhibit the activity of CYP enzymes [20], [21], [22], [23], [24], [25]. This inhibition can decrease warfarin clearance, leading to elevated plasma drug levels and, consequently, an increased INR in patients undergoing warfarin therapy [13], [14], [17], [25], [26]. The molecular mechanism underlying the interaction between warfarin and durian remains unclear. Therefore, this preliminary study aimed to provide initial insights into this interaction by bridging the gap between clinical practice and possible molecular mechanisms through metabolomic analysis using proton nuclear magnetic resonance (^1^H NMR).

## 2 Materials and methods

### 2.1 Ethical approval and informed consent

The study was conducted following the Declaration of Helsinki and received approval from the Institutional Review Board (Human Ethics Committee) of the Faculty of Medicine, Chiang Mai University, Thailand (ethics approval reference: 201/2023, Study code: FOR-2566–0144). Eligible patients were those on continuous warfarin therapy for more than six months who experienced a warfarin-durian interaction, resulting in an increased INR during the study period. All patients provided written informed consent before enrollment, and study results were reported anonymously.

### 2.2 Study design, setting, and population

This was a cross-sectional study conducted at a tertiary hospital in Thailand. The study included patients aged 18–70 on continuous warfarin therapy for more than six months and were followed at a warfarin clinic. Patients were divided into two groups: the case group and the control group. The case group consisted of those who consumed durian repeatedly (one to three pieces per day for a period ranging from five days to one month) while taking warfarin and subsequently experienced a drug interaction resulting in an increased INR. The control group consisted of patients who took warfarin without consuming durian and maintained an INR within the therapeutic range during the same period. All patients were closely monitored by physicians and pharmacists, with INR measured at every follow-up visit. Baseline INR was available for all patients from routine follow-up visits conducted at intervals ranging from 1 to 3 months, depending on individual patient stability. In all cases, INR consistently remained within the therapeutic range prior to the warfarin-durian interaction. Drug-food interactions were assessed by both a physician and a pharmacist. Patients were excluded if they were taking other anticoagulants, had acute or chronic infections, or were non-compliant.

### 2.3 Reagents and sample preparation

Methanol, chloroform, acetonitrile, deuterium oxide (D_2_O), and trimethylsilyl propanoic acid (TSP) were obtained from Sigma-Aldrich (St. Louis, MO, USA). All chemicals utilized in this study were of analytical grade. Plasma samples were mixed with acetonitrile (1:1) for ten minutes and then centrifuged at 4000 RPM. The supernatant was lyophilized and reconstituted in 0.6 mL of 0.1 M TSP in D_2_O. Metabolite concentrations were analyzed using 500 MHz NMR, following a previously optimized method to minimize water resonance interference [27], [28].

### 2.4 Acquisition parameters

^1^H NMR spectra were acquired using a Bruker AVANCE 500 MHz spectrometer (Bruker, Bremen, Germany) with a Carr–Purcell–Meiboom–Gill (CPMG, RD–90°, (t–180°), n–acquire) pulse sequence at 27 °C. Water signal suppression was achieved via pre-saturation. Key parameters included 16 scans, a one-second relaxation delay, a 3.95-second acquisition time, an 8278.146 Hz spectral width, and a 90° pulse with 16 signal averages (NSAs). Baseline and phase corrections were performed using TopSpin 4.0.7. Spectra (0–12 ppm) were normalized and analyzed using metabolomics databases, with TSP as an internal standard for quantifying 24 energy-related metabolites.

### 2.5 Internal standard (I.S.) and quality control (Q.C.)

TSP was chosen as the I.S. for its stable, reproducible signal at 0 ppm on a 500 MHz spectrometer. Its uniform proton environment ensures consistency, while its high-field positioning enables clear signal separation. Additionally, its chemical inertness and low boiling point allow efficient extraction. Q.C. samples were prepared by pooling equal portions of each sample to ensure calibration consistency and reduce variability in ^1^H NMR analysis. They followed the same procedures as test samples, adhering to established methodologies for non-targeted metabolite analysis.

### 2.6 Peak assignment, chemical identification, and ^1^H NMR data analysis

Chemical structures were identified using the Human Metabolome Database (HMDB, accessed March 1, 2024) [27], [28], [29], [30], [31]. Peak acquisition and *J*-coupling values were analyzed with Bruker TopSpin 4.0.7. NMR spectra interpretation relied on chemical shifts for signal identification, peak integration, spin-spin coupling, and pattern analysis. Non-target metabolite peaks were calibrated within 0.01 ppm of HMDB. Data export and spectral visualization were performed using MestRenova 12.0.0 (MestreLab Research, Santiago de Compostela, Spain), with median values reported and normality assessed via the Kolmogorov–Smirnov test [27], [28], [29], [30], [31].

### 2.7 Bioinformatics and statistical analysis

MetaboAnalyst (version 6.0, http://www.metaboanalyst.ca/MetaboAnalyst, accessed on March 1, 2024) was used for plasma metabolite analysis, pathway enrichment, pathway analysis, and metabolic profiling during the warfarin-durian interaction [30]. Heatmaps and Pearson correlation coefficients were generated to assess metabolic changes [30]. Key metabolites were evaluated as potential biomarkers using univariate receiver operating characteristic (ROC) curve analyses, with 95% confidence intervals (CIs) [30]. Demographic data were presented as frequencies (n, %) and means ± SD. The Mann-Whitney *U* test was applied to compare INR between case and control groups using GraphPad Prism (version 8.3.0, GraphPad Software, San Diego, California, USA, www.graphpad.com, accessed on March 1, 2024).

## 3 Results

Of the seven reported cases of warfarin-durian interactions, all individuals experienced increased INR following durian consumption. Patients reported consuming one to three pieces of durian per day for a duration ranging from five days to one month before the observed drug interaction. Other potential factors contributing to INR elevation, such as non-compliance, were not documented. The average INR in the warfarin-durian interaction group was 3.93 ± 0.60, compared to 2.48 ± 0.32 in the control group. This difference was statistically significant (*p* < 0.0001), suggesting that durian consumption may have influenced warfarin metabolism (Table 1).

**Table 1:** Demographic characteristics of patients.

**Variables**	**Warfarin-durian interaction** **(n = 7)**	**Control** **(n = 15)**
INR (Mean ± SD) (range)	3.93 ± 0.60* (3.10–5.00)	2.48 ± 0.32 (1.95–3.10)
Underlying disease requiring warfarin therapy (n, %) - Atrial fibrillation - Mitral valve replacement	6 (85.71) 1 (14.29)	12 (80.00) 3 (20.00)
Gender (n, %) - Male - Female	2 (28.57) 5 (71.43)	5 (33.33) 10 (66.67)

INR = international normalized ratio; * Mann-Whitney U Test (*p* < 0.0001)

**Fig. 1:**
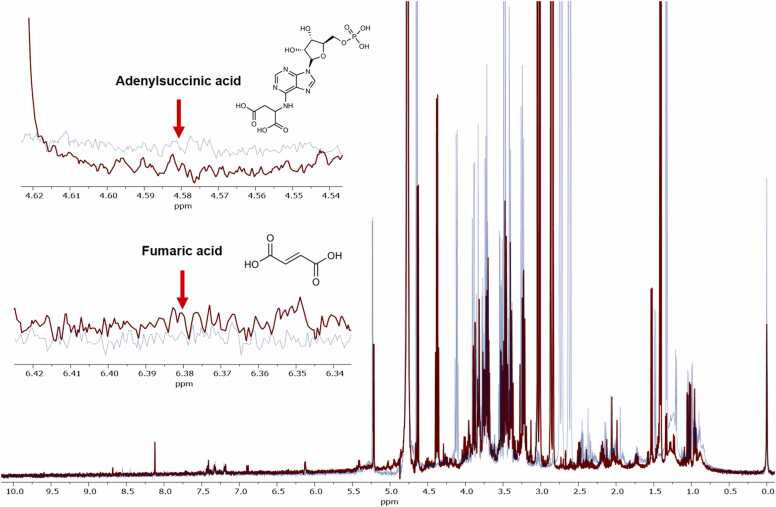
Results of proton nuclear magnetic resonance (^1^H NMR)-based metabolomics analysis. The 500 MHz ^1^H NMR spectra of plasma samples from individual patients are presented. The spectral overlay highlights distinct differences between samples collected from the warfarin-durian interaction group (red) compared with those from the control group (blue). Notably, a significant decrease in adenylsuccinic acid concentration (4.58 ppm) and an increase in fumaric acid concentration (6.38 ppm) were observed during the warfarin-durian interaction, as indicated by the arrows.

A 500 MHz ^1^H NMR spectrum of a plasma sample from an individual patient is shown in Fig. 1. The chromatogram overlay highlights differences between plasma samples collected during the warfarin-durian interaction compared to the control group. An untargeted metabolomics analysis identified 222 metabolites in both the warfarin-durian interaction and control groups. Metabolites were identified using the HMDB.

Sulfur-containing compounds are used as reliable biomarkers for durian exposure, providing confirmation in addition to patient-reported history. Several key exposure biomarkers were identified using the area under the ROC curve (AUROC) and their respective 95% CIs, T-statistics, and log2 fold change (FC), as demonstrated in Table 2 and Figures S2, S3, and S4. Specifically, hydrogen sulfide, trimethylsulfonium, and thiosulfate were significantly increased in the warfarin-durian interaction group compared to controls (*p* < 0.05). These findings underscore the potential utility of these sulfur-containing compounds as effective biomarkers for durian exposure.

**Table 2:** Receiver operating characteristic (ROC) curve analysis of exposure biomarkers.

**Biomarkers**	**AUROC**	**T-test**	**Log2 FC**
Hydrogen sulfide	1.0	1.6287E-9	2.2200
Trimethylsulfonium	0.96939	7.6332E-4	1.5091
Thiosulfate	0.88776	1.3789E-4	1.8590

AUROC = area under ROC curve; FC = fold change; ROC = Receiver Operating Characteristic

### 3.1 Analysis of related pathways

**Fig. 2:**
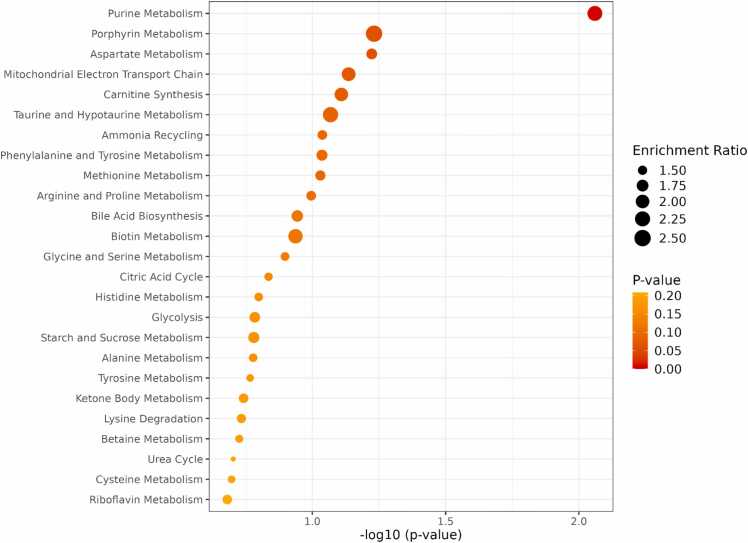
Pathway enrichment analysis revealed metabolic disruptions during the warfarin-durian interaction. A detailed visualization of the top 25 enriched metabolite sets, based on the pathway set library from the Small Molecule Pathway Database (SMPDB), displays both p-value significance and enrichment ratios. The high enrichment of purine metabolism suggests its critical role in the observed metabolic changes.

**Fig. 3:**
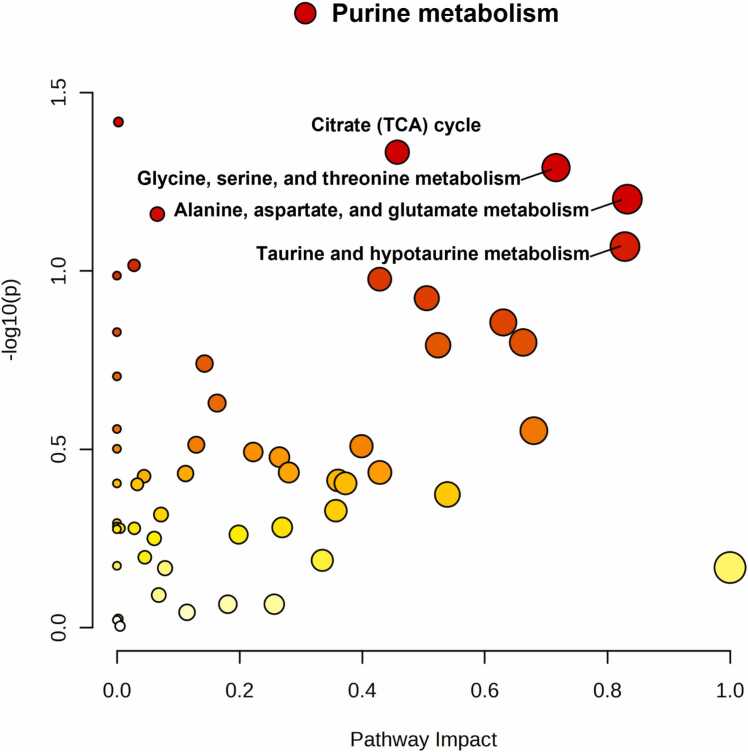
Pathway impact analysis identified significant metabolic disruptions, with purine metabolism exhibiting high statistical significance and high pathway impact. These findings suggest that purine metabolism plays a crucial role in the observed metabolic changes, which may contribute to elevated international normalized ratio (INR) and an increased risk of coagulation abnormalities.

Pathway enrichment and impact analysis was conducted using MetaboAnalyst (version 6.0). The analysis revealed significant metabolic disruptions between the warfarin-durian interaction group and the control group, as illustrated in Fig. 2, Fig. 3. Purine metabolism was identified as the only pathway that was significantly and highly impacted. These findings highlight the pivotal role of purine metabolism in the metabolic alterations associated with the warfarin-durian interaction. This interaction appears to affect purine metabolism, a series of biochemical reactions responsible for producing nucleotides essential for energy metabolism, DNA and RNA synthesis, and signal transduction [32]. These metabolic changes may contribute to elevated INR, suggesting an increased risk of coagulation abnormalities.

### 3.2 Alteration in metabolic profiles during Warfarin-Durian interaction

**Fig. 4:**
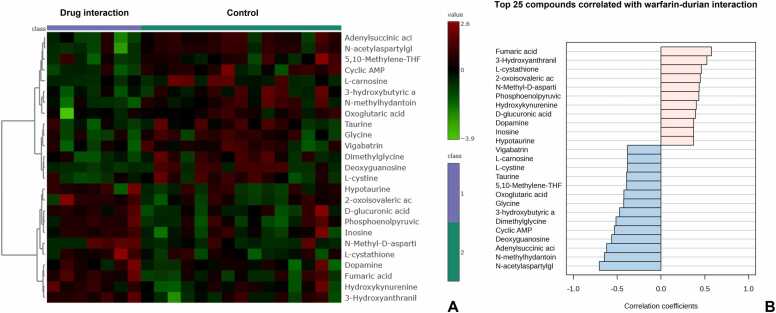
(A) Heatmaps illustrate the differential abundance of metabolites between the warfarin-durian interaction group and the control group, with red indicating higher intensities and green indicating lower intensities. (B) Bar graph depicting metabolic alterations, based on Pearson correlation coefficients assessing relationships among metabolites. This analysis highlights distinct patterns associated with the warfarin-durian interaction, aligning with trends observed in the heatmaps.

The heatmaps illustrate the differential abundance of metabolites between the warfarin-durian interaction group and the control group. In these visualizations, columns represent individual samples categorized into the two groups, while rows display the top 25 metabolites ranked by their correlation coefficients. Metabolite intensities are normalized and represented on a red-green color scale, where red denotes higher intensities and green denotes lower intensities (Fig. 4A).

To further explore metabolic alterations, Pearson correlation coefficients were calculated to examine relationships among metabolites, revealing distinct patterns associated with the warfarin-durian interaction. These correlations are presented in the bar graph (Fig. 4B), which aligns with trends observed in the heatmaps. Collectively, these findings indicate significant shifts in metabolite profiles, suggesting that the warfarin-durian interaction induces notable metabolic changes.

### 3.3 Key metabolites as potential biomarkers

**Fig. 5:**
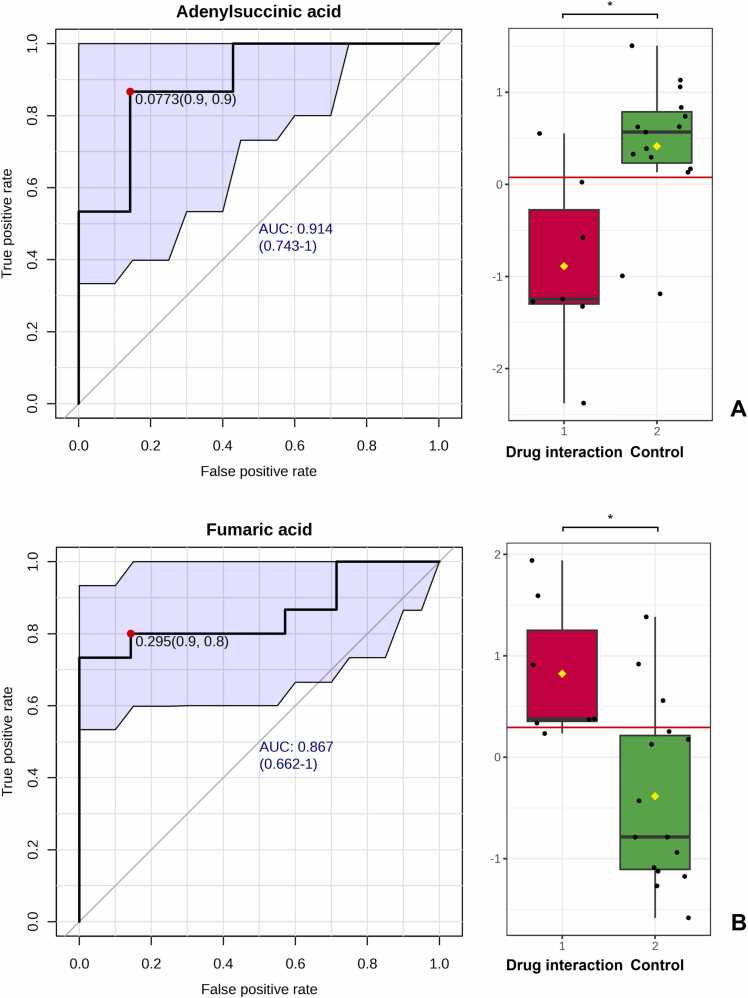
Classical univariate receiver operating characteristic (ROC) curve analysis and a bar graph showing the comparison of metabolite concentrations in the warfarin-durian interaction (red) and control (green) groups. (A) Adenylsuccinic acid, demonstrating an area under the ROC curve (AUROC) of 0.914 with 95% confidence intervals (CIs) of 0.743–1. A comparison of adenylsuccinic acid concentrations between the warfarin-durian interaction group (red) and the control group (green) indicates a significant reduction in adenylsuccinic acid in the warfarin-durian interaction group. (B) Fumaric acid, demonstrating an AUROC of 0.867 with 95% CIs of 0.662–1. A comparison of fumaric acid concentrations between the warfarin-durian interaction group (red) and the control group (green) indicates a significant increase in fumaric acid in the warfarin-durian interaction group.

Classical univariate ROC curve analyses were performed to assess each biomarker for the AUROC and their respective 95% CIs. The biomarkers were ranked based on AUROC, T-statistics, and log2 FC, as shown in Table 3. Key metabolites were selected based on the statistically significant differences in their concentrations between the warfarin-durian interaction group and the control group. The analysis identified the top 15 significant metabolites, including n-acetylaspartylglutamic acid, n-methylhydantoin, adenylsuccinic acid, fumaric acid, deoxyguanosine, cyclic adenosine monophosphate (cAMP), 3-hydroxyanthranilic acid, dimethylglycine, 3-hydroxybutyric acid, l-cystathione, 2-oxoisovaleric acid, n-methyl-d-aspartic acid, phosphoenolpyruvic acid, glycine, and oxoglutaric acid. Two biomarkers were selected for further analysis: adenylsuccinic acid and fumaric acid. Adenylsuccinic acid exhibited a high AUROC of 0.89524, indicating its potential as an effective biomarker (*p* = 0.0024). Fumaric acid showed an AUROC of 0.85714, also indicating its potential (*p* = 0.0051), as demonstrated in Table 3 and Fig. 5.

**Table 3:** Receiver operating characteristic (ROC) curve analysis of individual biomarkers.

**Biomarkers**	**AUROC**	**T-test**	**Log2 FC**
N-acetylaspartylglutamic acid	0.90476	0.000263	-0.47054
N-methylhydantoin	0.91429	0.001136	-0.00612
Adenylsuccinic acid	0.89524	0.002040	-0.34558
Fumaric acid	0.85714	0.005065	0.77303
Deoxyguanosine	0.81905	0.006467	-2.61810
Cyclic adenosine monophosphate (cAMP)	0.82857	0.010864	-0.44180
3-hydroxyanthranilic acid	0.84762	0.012269	0.91676
Dimethylglycine	0.80000	0.014393	-0.37560
3-hydroxybutyric acid	0.74286	0.026401	0.09923
L-cystathione	0.76190	0.031148	0.74705
2-oxoisovaleric acid	0.78095	0.036086	0.61363
N-methyl-D-aspartic acid	0.70476	0.042675	1.88110
Phosphoenolpyruvic acid	0.79048	0.044632	0.62716
Glycine	0.76190	0.048954	-0.65415
Oxoglutaric acid	0.74286	0.049459	-0.45716

AUROC = area under ROC curve; FC = fold change; ROC = Receiver Operating Characteristic

## 4 Discussion

Purine metabolism plays an essential role in platelet activation by generating key signaling molecules that regulate thrombus formation [33]. The catabolism of adenosine triphosphate (ATP) leads to the production of adenosine diphosphate (ADP), adenosine monophosphate (AMP), and adenosine (ADO), which are further hydrolyzed by specific ectoenzymes [34]. Among these purine-derived metabolites, ADP serves as a potent activator of platelet aggregation [33], [35]. Upon release, ADP binds to P2Y1 and P2Y12 receptors, after which P2Y1 activation stimulates phospholipase C, leading to platelet shape changes and the initiation of aggregation [36], [37]. Simultaneously, P2Y12 receptor activation inhibits cAMP production, facilitating full platelet activation and stabilizing the platelet aggregate [37]. The interplay between these receptors amplifies platelet recruitment and enhances thrombus formation at the site of vascular injury, contributing to effective hemostatic function [37], [38].

Recent evidence from venous thrombosis models suggests that purine metabolism plays a pivotal role in thrombus formation and progression [39]. Metabolic profiling of thrombi has revealed a significant accumulation of AMP, guanosine monophosphate (GMP), hypoxanthine, and guanine compared to their levels in circulating blood [39]. This enrichment is indicative of enhanced purine nucleotide catabolism, which is closely associated with the hypoxic and metabolically altered microenvironment within venous thrombi [39]. Metabolomic studies have identified alterations in glycolytic, purine, and redox-related metabolites, which are particularly prominent in erythrocyte-rich thrombi [40]. Furthermore, in patients with high-risk pulmonary embolism (PE), significant reductions in tricarboxylic acid (TCA) cycle intermediates—including alpha-ketoglutarate, malate, isocitrate, fumarate, and cis-aconitate—have been observed [39]. This reduction suggests a metabolic shift favoring glycolysis, as evidenced by concomitant increases in pyruvate and lactate concentrations [39]. Notably, elevated levels of hypoxanthine and xanthosine have been detected in patients with intermediate- and high-risk PE, underscoring the role of ATP breakdown under hypoxic conditions as a driving force behind purine metabolite accumulation [39]. Beyond ATP degradation, the regulation of purine flux in thrombotic environments is further modulated by erythrocyte-mediated uptake and release of purines, which respond dynamically to changes in pH, inorganic phosphate levels, and oxygen tension [39], [41]. These findings collectively suggest that disruptions in purine metabolism may serve as a hallmark of thrombus formation and progression, offering potential metabolic targets for therapeutic intervention in thrombosis-related disorders [39], [41].

**Fig. 6:**
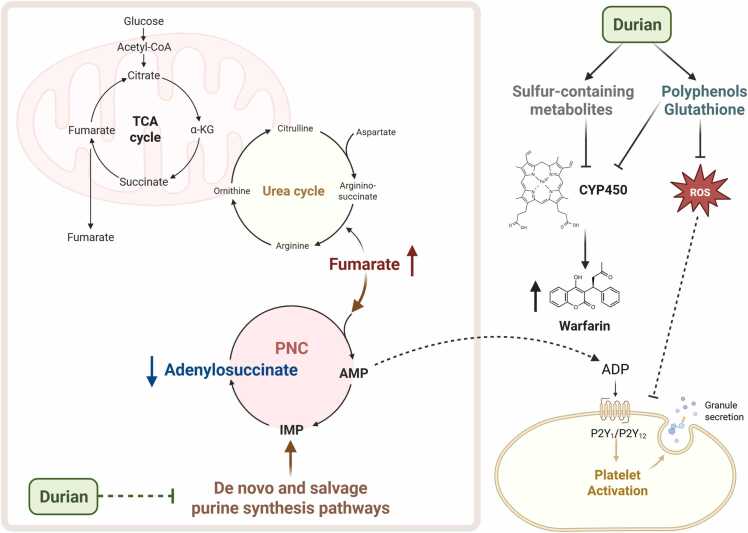
The proposed mechanism of warfarin and durian interaction illustrates the disruption of purine metabolism and the tricarboxylic acid (TCA) cycle. Adenylosuccinic acid and fumaric acid are highlighted as key metabolic biomarkers. A decline in adenylosuccinic acid and an increase in fumaric acid are associated with metabolic dysregulation, which may result in elevated international normalized ratio (INR) in patients. ADP, adenosine diphosphate; AMP, adenosine monophosphate; CYP450, cytochrome P450; IMP, inosine monophosphate; PNC, purine nucleotide cycle; ROS, reactive oxygen species; TCA, tricarboxylic acid.

Metabolic profiling using ^1^H NMR analysis identified potential biomarkers associated with warfarin-durian interactions, including reduced adenylsuccinic acid and increased fumaric acid (fumarate). These findings suggest disruptions in purine metabolism, including both the de novo purine synthesis and salvage pathways, as well as their interplay with fumarate in the TCA cycle [42], [43]. Adenylsuccinic acid or adenylosuccinate (S-AMP) is an intermediate metabolite in the biosynthesis of AMP from inosine monophosphate (IMP) [42], [43], [44]. Its reduction during the warfarin-durian interaction suggests a disruption in purine metabolism, leading to an enhanced conversion of S-AMP to AMP [42], [43], [44]. This conversion process is accompanied by the release of fumarate as a byproduct, catalyzed by adenylosuccinate lyase [42], [43]. The observed increase in fumarate further supports the hypothesis of an accelerated S-AMP-to-AMP conversion during the warfarin-durian interaction [42], [43]. Additionally, the purine metabolism pathway is interconnected with the TCA cycle via fumarate, which is produced during the purine nucleotide cycle (PNC) [45], [46]. Fumarate directly enters the TCA cycle, supporting metabolic flux and ATP production [45], [46]. As a result, purine metabolism provides carbon skeletons to the TCA cycle, playing a crucial role in cellular energy homeostasis [45], [46]. Our findings indicate that the decrease in S-AMP, an intermediate metabolite in purine metabolism, reflects a disruption in this pathway [42], [43], [44]. This disruption may impair platelet function and potentially synergize with the anticoagulant effects of warfarin, leading to elevated INR in patients [33], [35]. Furthermore, the observed increase in fumarate in patients with elevated INR aligns with previous studies, which have reported significant reductions in TCA cycle intermediates, including fumarate, in patients with high-risk PE [39], [41]. This suggests that disruptions in both purine metabolism and the TCA cycle may contribute to the underlying mechanism of the warfarin-durian interaction (Fig. 6).

Durian (*Durio zibethinus* M.) is rich in polyphenols, a major class of antioxidants, play a pivotal role in alleviating oxidative stress by neutralizing reactive oxygen species (ROS) [26]. In addition, durian contains other potent antioxidants, including γ-glutamylcysteine and glutathione [47]. Excessive ROS levels can initiate platelet activation through pathways such as nicotinamide adenine dinucleotide phosphate (NADPH) oxidase and mitochondrial ROS production [48], [49]. This process leads to platelet aggregation, adhesion, and recruitment—key events in thrombus formation [48], [49]. Moreover, ROS contribute to a procoagulant platelet phenotype, enhancing their clot-forming potential, and compromising red blood cell membrane integrity and function, thereby promoting a hypercoagulable state [50]. Additionally, previous studies have demonstrated that glutathione can modulate coagulation in a concentration-dependent manner, leading to prolongation of activated partial thromboplastin time (APTT), prothrombin time (PT), and thrombin time (TT), as well as reduction in fibrinogen (FIB) levels in plasma [51]. According to several studies, glutathione and other antioxidants present in durian may influence hemostatic processes [48], [49].

Another interesting mechanism related to our findings on exposure biomarkers is that durian pulp contains sulfur-based compounds, which may potentially interfere with the activity of CYP metabolizing enzymes [20], [21], [22], [23], [24], [25], [26]. Silymarin components and their sulfate conjugates have been shown to significantly inhibit CYP2C9, CYP2C19, and/or CYP3A4, the primary enzymes responsible for warfarin metabolism [20]. Furthermore, these compounds have been found to substantially displace warfarin from the albumin protein [20]. Additionally, diallyl sulfide, a compound found in garlic, has been shown to inhibit CYP2E1 [24]. The activities of CYP metabolizing enzymes, particularly CYP1A2 and CYP2C9, have also been reported to be inhibited in rats with acute H₂S poisoning [22]. Moreover, both *in vitro* and clinical studies have provided evidence that sulfa drugs, such as sulfinpyrazone and cotrimoxazole, interact with warfarin by interfering with CYP2C9 activity [23], [25]. Several studies support our finding that the significant increase in sulfur-containing metabolites such as hydrogen sulfide, trimethylsulfonium, and thiosulfate in the warfarin-durian interaction group may be indicative of altered CYP enzyme activity and an enhanced potential for fruit-drug interactions.

To date, this preliminary study represents the first application of ^1^H NMR metabolomics analysis to explore possible molecular mechanisms underlying the effects of durian consumption on warfarin efficacy, based on clinical observations of INR. Our findings indicate that the warfarin-durian interaction perturbs purine metabolism and the TCA cycle, with adenylosuccinic acid and fumaric acid identified as potential metabolic biomarkers. Specifically, the observed reduction in adenylosuccinic acid and elevation in fumaric acid may contribute to dysregulation of purine metabolism and the TCA cycle, potentially leading to an increase in INR. Further investigations are warranted to delineate the precise biochemical pathways involved. To enhance the robustness and generalizability of these findings, future studies should incorporate larger sample sizes and be conducted across multiple centers. The application of ^1^H NMR metabolomics analysis remains a valuable approach for advancing the understanding of complicated drug interactions, as demonstrated by previous research [27], [52].

## 5 Conclusions

This preliminary study is the first to apply ^1^H NMR metabolomics analysis to investigate the possible molecular mechanisms underlying drug interactions, focused on the interaction between warfarin and durian. By integrating clinical outcomes with metabolic insights, our findings indicate that this interaction disrupts purine metabolism and the TCA cycle, with adenylosuccinic acid and fumaric acid identified as potential metabolic biomarkers. Specifically, the observed decline in adenylosuccinic acid and the increase in fumaric acid may contribute to metabolic dysregulation, potentially leading to an elevation in INR. Interestingly, this study used sulfur-containing compounds as biomarkers of durian exposure, thereby providing confirmation in addition to patient-reported history. Our findings indicate that the concentrations of hydrogen sulfide, trimethylsulfonium, and thiosulfate were significantly elevated in the warfarin-durian interaction group. The application of ^1^H NMR metabolomics for monitoring metabolic alterations during the warfarin-durian interaction provides a valuable approach for acquiring deeper insights into drug interactions and their physiological consequences.

## CRediT authorship contribution statement

**Churdsak Jaikang:** Validation, Supervision, Formal analysis, Conceptualization. **Preechaya Tajai:** Writing – review & editing, Writing – original draft, Visualization, Validation, Supervision, Software, Resources, Project administration, Methodology, Investigation, Funding acquisition, Formal analysis, Data curation, Conceptualization. **Konwalin Wannaphueak:** Writing – original draft, Software, Investigation, Formal analysis, Data curation. **Piyapat Rattanasuwan:** Writing – original draft, Software, Investigation, Formal analysis, Data curation. **Prem Lertpongpipat:** Writing – original draft, Software, Investigation, Formal analysis, Data curation. **Giatgong Konguthaithip:** Software, Investigation, Formal analysis, Data curation. **Natthapat Hiranchatchawal:** Writing – original draft, Software, Investigation, Formal analysis, Data curation.

## Declaration of Competing Interest

The authors declare that they have no known competing financial interests or personal relationships that could have appeared to influence the work reported in this paper.

## Data Availability

Data will be made available on request.
