# Supplementary material for: Warfarin-Durian interaction: A case study bridging clinical outcomes and metabolic profiling through metabolomics
Source: Toxicol Rep. 2026 Feb 25;16:102232. doi: 10.1016/j.toxrep.2026.102232 (PMC12963912; doi:10.1016/j.toxrep.2026.102232)
Supplement: Supplementary file 1 — Supplementary material [file mmc1.docx]

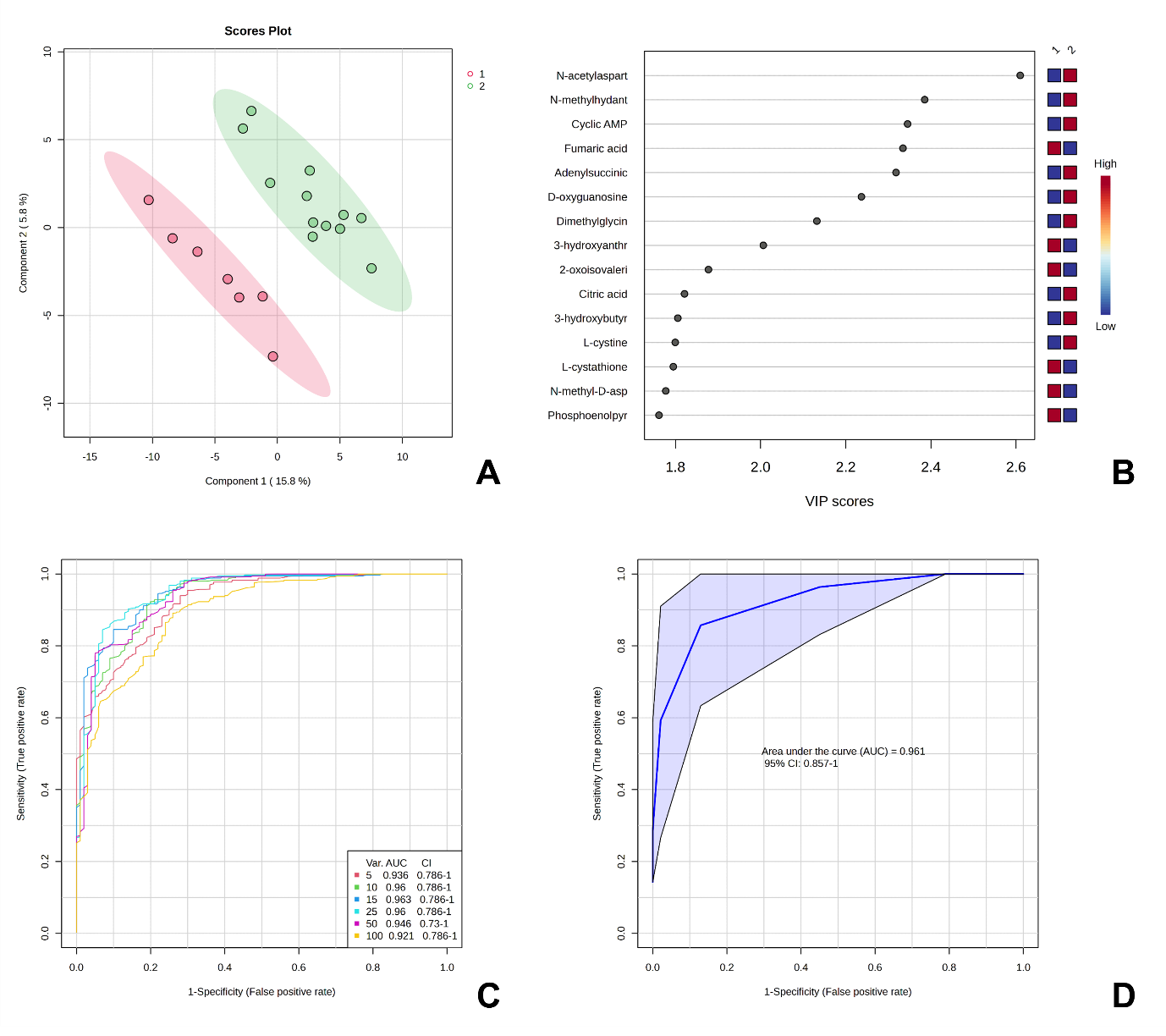


**Figure S1.** (A) The partial least squares discriminant analysis (PLS-DA) scores plot illustrates a clear distinction between the two groups: the warfarin-durian interaction group (group 1, displayed in red) and the control group (group 2, displayed in green), indicating a significant difference in their metabolic profiles. (B) The variable importance in the projection (VIP) plot shows the metabolites selected by the PLS-DA model, ranked according to their VIP scores, representing their relative importance. The top ten metabolites include N-acetylaspartate, N-methylhydantoin, cyclic adenosine monophosphate (cAMP), fumaric acid, adenylsuccinic acid, deoxyguanosine, dimethylglycine, 3-hydroxyanthranilic acid, 2-oxovaleric acid, and citric acid. (C) Multivariate exploratory receiver operating characteristic (ROC) analysis, based on cross-validation (CV) performance averaged across all models and CV runs, assesses the model’s diagnostic accuracy. (D) An area under the curve (AUC) of 0.961 indicates a strong ability to differentiate between the groups, with a 95% confidence interval (CIs) of 0.857–1.


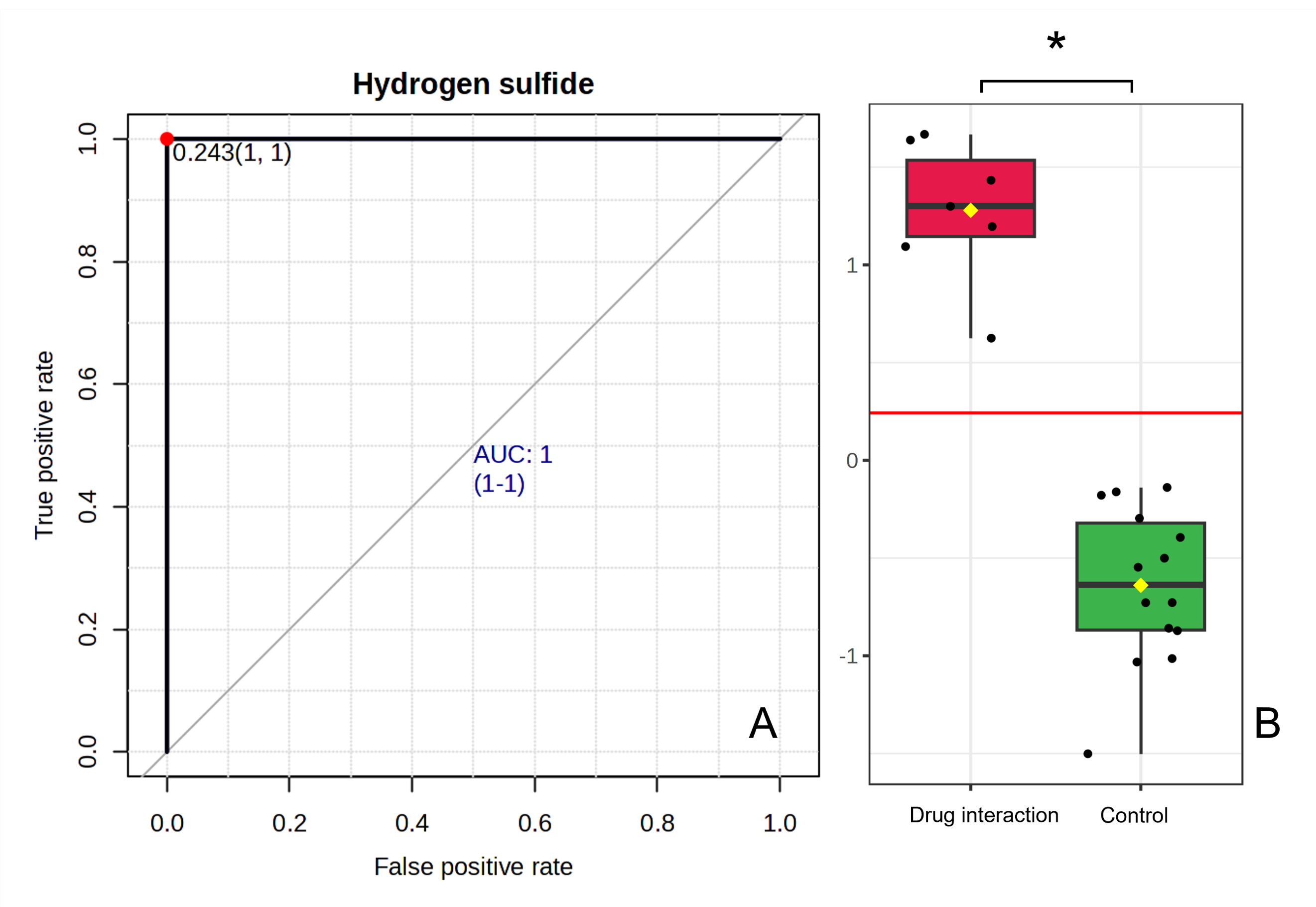


**Figure S2.** Classical univariate receiver operating characteristic (ROC) curve analysis and a bar graph showing the comparison of metabolite concentrations between the warfarin-durian interaction (red) and control (green) groups. (A) Hydrogen sulfide, demonstrating an area under the ROC curve (AUROC) of 1.000 with 95% confidence intervals (CIs) of 1–1. (B) A comparison of hydrogen sulfide concentrations between the warfarin-durian interaction group (red) and the control group (green) indicates a significant increase in the warfarin-durian interaction group.


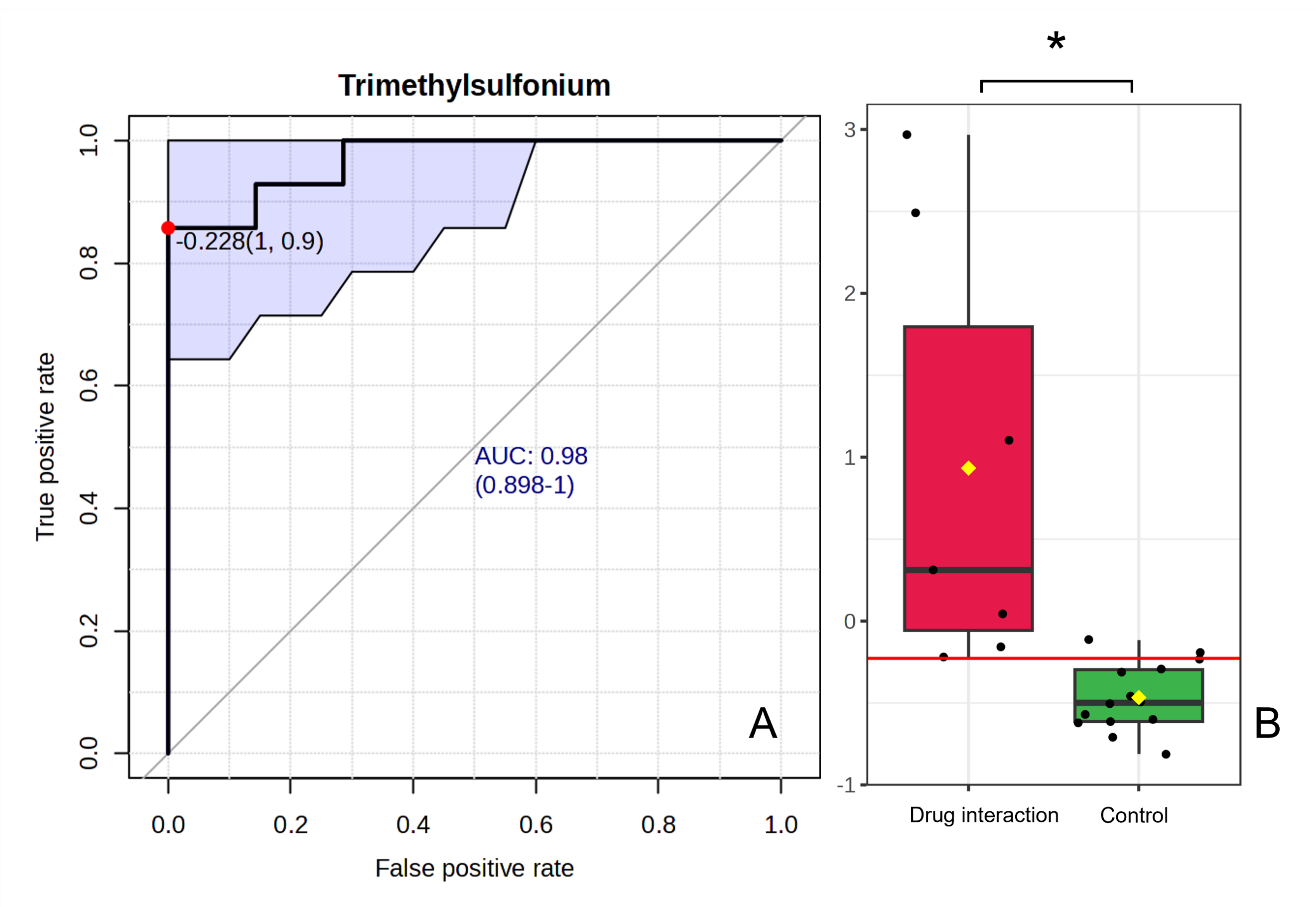


**Figure S3.** Classical univariate receiver operating characteristic (ROC) curve analysis and a bar graph comparing metabolite concentrations between the warfarin-durian interaction (red) and control (green) groups. (A) Trimethylsulfonium, showing an area under the ROC curve (AUROC) of 0.980 with 95% confidence intervals (CIs) of 0.898–1. (B) Comparison of trimethylsulfonium concentrations between the warfarin-durian interaction group (red) and the control group (green) indicates a significant increase in the warfarin-durian interaction group.


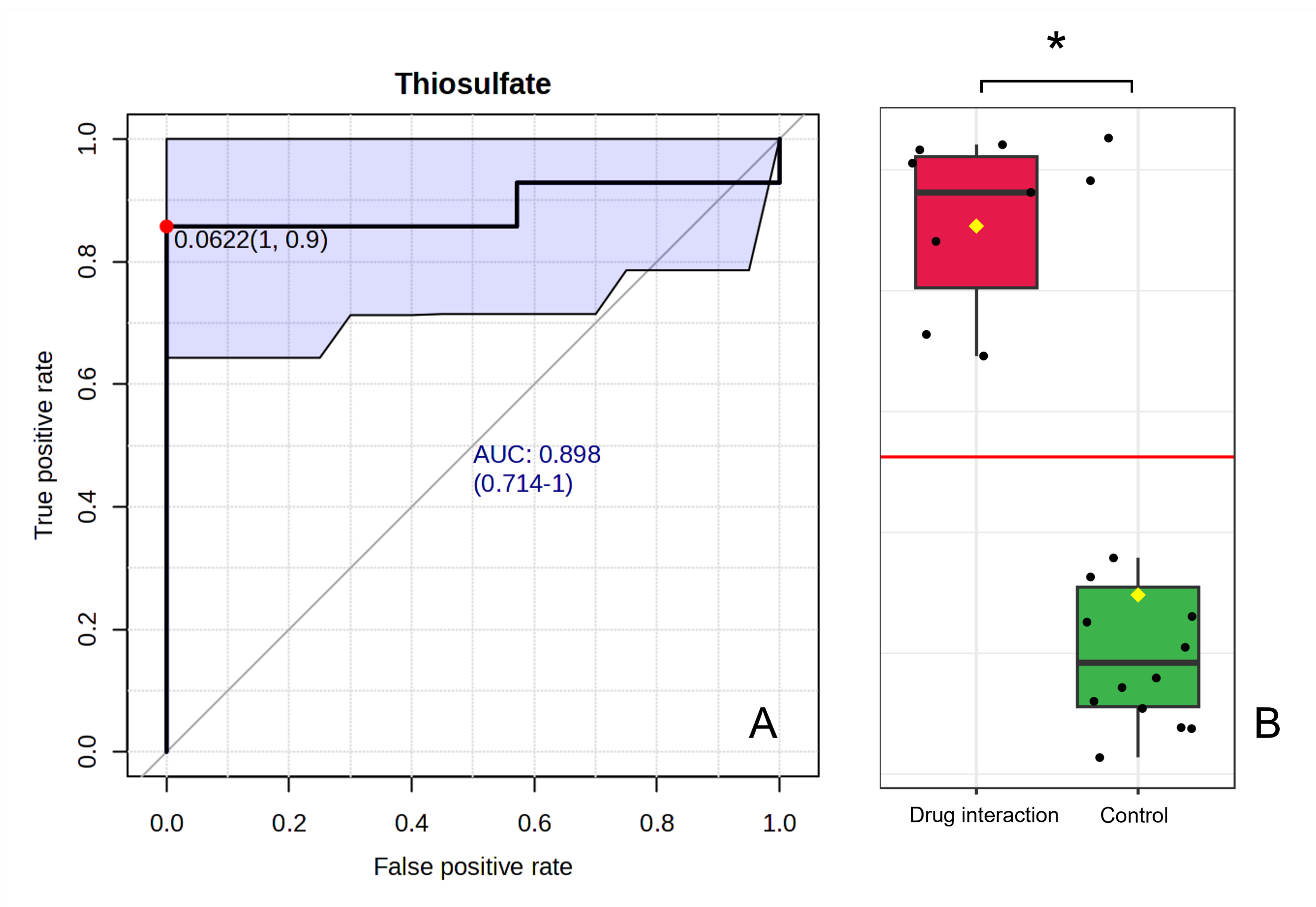


**Figure S4.** Classical univariate receiver operating characteristic (ROC) curve analysis and a bar graph comparing metabolite concentrations between the warfarin-durian interaction (red) and control (green) groups. (A) Thiosulfate, showing an area under the ROC curve (AUROC) of 0.898 with 95% confidence intervals (CIs) of 0.714–1. (B) Comparison of thiosulfate concentrations between the warfarin-durian interaction group (red) and the control group (green) indicates a significant increase in the warfarin-durian interaction group.
